# Oral health behavior among school children aged 11–13 years in Saveh, Iran: an evaluation of a theory-driven intervention

**DOI:** 10.1186/s12887-020-02381-6

**Published:** 2020-10-13

**Authors:** Mahmood Karimy, Peter Higgs, Shaghaygh Solayman Abadi, Bahram Armoon, Marzieh Araban, Mohammad Reza Rouhani, Fereshteh Zamani-Alavijeh

**Affiliations:** 1 Department of Public Health, Faculty of Health, Social Determinants of Health Research Center, Saveh University of medical sciences, Saveh, Iran; 2 Department of Public Health, School of Psychology and Public Health, La Trobe University, Melbourne, Australia; 3 Department of health education and promotion, Social Determinants of Health Research Center, Ahvaz Jundishapur University of Medical Sciences, Ahvaz, Iran; 4 Internal medicine department, faculty of medicine, Arak University of medical sciences, Arak, Iran; 5 Department of health education and promotion, School of Health, Isfahan University of Medical Sciences, Isfahan, Iran

**Keywords:** Oral health behavior, Children, Peer-led education, Theory of planned behavior

## Abstract

**Background:**

Good oral health (OH) is essential for physical, social, mental health, and overall quality of life. This study assessed the usefulness of the theory of planned behavior (TPB) in changing oral health-related behaviors among school children aged 11–13 years in Saveh, Iran.

**Methods:**

In this descriptive before and after study, participants were sixth-grade students at single sex primary schools in Saveh city, Iran. We recruited 356 school children in 2019. Using simple random sampling, a male and a female school per district were allocated to the experimental group and the remaining schools to the control group. Our planned oral health education consisted of four one-hour training sessions over 1 week. The first session familiarized the participants with important information about OH. In the second session, we applied a brain storming exercise to identify the benefits and barriers to flossing and brushing. In the third session, a short film about correct brushing and dental flossing technique was shown and research team also used role-playing to correct any mistakes. In the final session students were taught about the importance and the application of OH planning and given forms to help plan for brushing.

**Results:**

Participants for the study included 356 students (180 in the experimental group and 176 in the control group) who completed the post-test questionnaire. The mean age ± standard deviation was 11.55 years ±0.93 in the experimental group and 11.58 years ±1.01 in the control group. After the intervention, the paired t-test indicated a significant difference between the mean and standard deviation of the action plan and coping plan constructs in the experimental group before and after the intervention (*p* < 0.05). Covariance analysis indicated a significant difference between scores of intervention and control groups under statistical control of post-test in two groups (covariate) after a peer-led education program (post-test) (*p <* 0.05).

**Conclusion:**

A shortage of professional health workers in education settings together with the ease, usefulness and low-cost of this peer-led method, suggest further steps should be taken to implement it more widely to improve and enhance primary school aged students’ oral health behavior.

## Background

Oral health (OH) is an important component of overall human health and is inextricably linked with physical [1], social and mental health, and quality of life [2, 3]. In children, poor oral hygiene and untreated oral disease have a devastating impact on individual growth and development [4]. According to the World Health Organization (WHO), poor OH can have a negative effect on children’s performance at school and impacts their future academic success. Oral health problems also reduce people’s ability to smile, eat, and talk, and have a detrimental effect upon their social and mental health [5]. Tooth decay and gum disease are common affecting more than 26% of school aged children globally [6]. Over 66 million school hours per year are lost due to OH-related problems [7]. Oral disease is also more prevalent among children in developing countries compared to developed ones [8]. For instance, the prevalence of dental caries is 53% among four-year-old children in countries such as India and China, and 40% in South Africa [9], while it is 32% in England and 22% in Italy [10]. While the WHO has set an ambitious goal to reduce the mean decayed, missing, and filled teeth (DMFT) index to less than one [4] a recent study in Kurdistan province Iran, found the DMTF in 2000 people aged 15–40 to be 7.33 ± 3.0 [11]. As children are highly susceptible to tooth decay, investigating dental caries indexes and understanding the factors affecting them is an important public health goal.

Iranian children aged between 12 and 14 years are very susceptible to oral diseases [12]. Previous research has found that the mean DMFT index in Iranian primary school children ranged between 3.54 and 4.2 [13]. About one fifth of 2 to 4- year-old children have caries which dentists can identify, and by age 17, approximately 80% of young people have at least one decayed tooth [14, 15]. Most prevalence studies conducted over the past 10 years in Iran have reported dental caries in between 35 and 85% of their participants [16, 17].

Choosing and finding effective, inexpensive, accessible, and inclusive educational solutions for improving oral health among adolescents is a concern of health policymakers [18]. Dental professionals believe a key to reducing caries and dental disease is to focus on prevention, and the first step in prevention is to raise awareness [19]. Health education is therefore key to preventing oral disease and promoting social health [20]. Peer-led education approaches are an effective training method as information has been found to be easily transmitted and understood by peers [21]. Peer education has also been found to be an effective strategy in supporting behavior change [22].

The theory of planned behavior (TPB) provides a good framework for understanding health education and has been applied previously in oral health settings [23]. TPB can be used alone as a theoretical basis for a health education program, and it can also be used in combination with other learning theories and approaches [24]. Despite previous meta-analysis having established TPB as the most valid theory for predicting, describing, and understanding OH behavior [23] there are also limitations to using TPB including a gap between the intention and action of participants [23, 25, 26].

TPB is a social cognition model of decision-making which supposes that social behavior is planned and that it accounts for the consequences of the behavior. Intention, is affected by attitude towards the behaviour (the degree to which performance of the behaviour is assessed as positive), subjective norms (perceived social agreement for participating in a behaviour), and perceived behavioural control (PBC); understanding of their controllability and capability to carry out a given behavior [27].

A meta-analysis study of 237 prospective tests of the TPB reported that intention and PBC together have an average of 19.3% of the variance in behaviour and 44.3% of the variation in intention [28]. To resolve this gap between “intention-behaviour” the development of action plans when, where and how to perform tasks can increase the probability of successful intention implementation [29].

Previous research on oral health behaviours found about half the individuals who wanted to perform certain oral health behaviours actually practice them [30]. Advance planning enables a mental association between specific situational cues and behavioural reactions, improving the availability of pertaining cues and reducing the likelihood of missing of opportunities to change behaviour [31]. There are two types of planning: action planning (defining when, where and how to act) and coping planning (planning how to deal with barriers to the intended behavior [32]. Action planning defines when, where and how to act and coping planning is a barrier-focused, distraction-inhibiting, self-regulation strategy maintaining goal-directed behaviour which mentally associates expected risk situations to appropriate coping responses [32]. Several studies have demonstrated useful effects of action planning and coping planning on behaviour, such as dental flossing [33, 34] and other health and self-care behaviours [35, 36]. While planning is most likely to work in situations where the intentions are high, evidence from across various oral and other selfcare behaviours does not necessarily support this assumption [36].

Advance planning establishes a mental relationship between specific situational signs and behavioural reactions, improving the availability of relevant signs and reducing the probability of missing of suitable opportunities [30]. Two types of planning have been recognized; action planning (AP) and coping planning (CP) [6]. Action planning determines when, where and how to act and coping planning notices to the barriers, distraction-inhibiting, self-regulation strategy achieving goal-directed behavior which mentally relates to risk [32].

The theoretical basis of the present study extended TPB to include the constructs CP and AP. While some studies have proven the effectiveness of peer-led education [37–39], other studies have not found significant differences between the scores of a peer group and those of a control group after the peer-led educational intervention [40, 41]. Due to the limited number of theory-based studies using a peer-led education approach, the present study was designed to assess the impact of an oral health educational intervention by using a peer-led education approach based on the extended TPB framework which sought to improve primary school aged students OH behavior.

## Methods

### Study design

In this descriptive before and after study, the research population consisted of sixth-grade students at primary schools in Saveh city, northwest Iran. All schools in Iran are single sex. We have used the STROBE checklist in the design of this study. (Additional file 1).

### Sampling and sample size

The sample size was determined to be 159 students per group (experimental and control groups) according to a formula based on Shiran’s study [42] with α = 0.05, ß = 0.1, and the effect size = 0.5. Anticipating up to 20% loss, 200 individuals were selected for the experimental group and 180 for the control group. In the experimental group, 20 individuals were included in the study to be recruited as the peer-leaders for the education group. The students were selected through multistage sampling from across sixth-grade primary schools. To this end, a list of all schools was prepared based on municipal districts (2 districts) in Saveh. Then, four schools per district (2 females and two male schools) and a total of 8 schools from 2 districts were selected. Using simple random sampling, a male and a female school per district were allocated into the experimental group and the remaining schools were placed in the control group. All sixth-grade students of the selected schools were eligible to be included in the study.

### Inclusion and exclusion criteria

Study participants were in the sixth grade of primary school and written consent from parents and students was required to be included in the study. Children with previous oral health education, those who were unable to brush and use dental floss due to physical problems; and those who missed more than one session during the period were excluded.

### Data collection and measurement

The data collection tool for the present study was a multi-part questionnaire: First, demographic characteristics (10 questions); second, attitudes (11 questions) with a 5-point Likert scale from 1 (strongly disagree) to 5 (strongly agree). Third, subjective norms were measured with four questions that could be answered according to a range of 1 (it is never true) to 5 (it is always true). Fourth, measuring perceived behavioral control (five questions) on a 5-point Likert scale from 1 (strongly disagree) to 5 (strongly agree). The fifth, behavioral intentions (four questions) answered on a 5-point Likert scale from 5 (very likely) to 1 (very unlikely). Sixth, action planning with four open-ended questions about the time, place, and duration of tooth brushing. Seventh, coping planning with two questions, including the barriers preventing them from carrying out their plans (brushing regularly), and also “how I can resolve this problem?”, and eighth, behavior with two questions about frequency of tooth brushing and dental flossing. English language version of the questionnaire was uploaded as additional file. (Additional file 2).

The tools in the present study were evaluated and validated by eight faculty members of Saveh University of Medical Sciences. The reliability of the questionnaire was confirmed by the internal consistency method for the whole instrument (Cronbach’s α = 0.85).

### Procedure

After identifying the target population and before the intervention, briefings were held for teachers and students in the presence of the main researcher who provided all information about the study. At the same session, the pre-test and peer group selection questionnaires were distributed. A list of student names in the class was given to participants and they were asked to name three class members, who they would like to teach in the priority order (1, 2 and 3). After group assessment, selected students were interviewed to evaluate their interest as well as their suitability for leading the peer component of the intervention. At this stage, 19 students were selected; (about one in every ten students) as the teaching interface for oral health to their friends.

### Intervention

The students received oral health education consisting of four one-hour training sessions over 1 week. The first session familiarized them with important information about OH. The session was followed by a brief introduction of oral structure, stages of permanent tooth eruption, dental caries, and causes of dental caries, gum and its diseases, and methods of preventing the oral disease. The peer-led education was provided through slides and photos. In the second session, brain storming was used and students were encouraged to think about any possible benefits and barriers to flossing and brushing. The role and importance of the use of dental floss and toothbrushes and their positive effects on OH were then emphasized by group discussion. At the beginning of the third session, a short film outlining correct brushing and dental flossing technique was shown. The research team also used role-playing for learning and feedback was provided as the mechanism for correcting mistakes. In the fourth (final) session students were taught about the importance of OH planning and how to apply it. The students were provided with a form containing four questions in which students were asked to write a plan for brushing (place, time, and duration of brushing). Students were instructed to place the forms where they would be visible and to follow the planned behavior written on the forms. Separately, participants were asked two questions about ways of overcoming any problems and to outline any obstacles to their program (plans to cope with them), and the ways to resolve this problem.

After the end of the 4 training sessions, the peer group was asked to run a similar training for ten students under its supervision for 1 week. During this training program, a research team supervised the implementation. The control group received the routine 4-week training program, but they received no extra training from the research team or the peer group. The self-administered questionnaires were completed by students in both arms of the study 2 months after the last educational session in order to evaluate the training results.

### Statistical analysis

Data analysis was done by SPSS 18; the paired sample t-test was used to determine attitude toward OH, PBC about OH, SN, AP, and CP within the intervention group by comparing the before and after intervention scores. A similar analysis was done for the control group. The independent-sample t-test was used to determine the effect of the intervention by comparing participants brushing and dental flossing scores between the intervention and control groups. Moreover, univariate analyses of covariance (ANCOVA) are computed with oral health behavior as dependent variables, TPB constructs as covariates.

## Results

Participants included 356 students (175 boys and 181 girls), 180 in the experimental group and 176 in the control group who completed the post-test questionnaire. The mean age ± standard deviation was 11.55 years ±0.93 in the experimental group and 11.58 years ±1.01 in the control group. Fathers literacy levels in the experimental and control groups was generally high with over 60% (*N* = 228) reporting at least high school education. For mothers almost half (45%) had at least completed a high school education. Few students (*N* = 14) reported their families were financially unstable. Both groups were similar in terms of demographic variables (see Table 1).

**Table 1:** Comparison of demographic variables between the both groups

Variables	Experimental		Control		***P***-value
	Number	Percentage (%)	Number	Percentage (%)	
**Sex**					
Boy	90	50	85	48.3	0.74
Girl	90	50	91	51.7	
**economic**					
good	107	59.5	101	57.4	
medium	65	36.1	69	39.2	0.63
weak	8	4.4	6	3.4	
**Mother’s job**					
Housewife	135	0.75	128	72.7	0.60
Others	45	0.25	48	27.3	
**Father’s education**					
Illiterate	8	4.4	10	5.7	0.95
Elementary	26	14.5	23	13.1	
Middle school	34	18.9	31	17.6	
High school and diploma	59	32.8	61	34.6	
Academic	53	29.4	51	29	
**Mother’s education**					
Illiterate	10	5.6	11	6.2	0.78
Elementary	30	16.6	38	21.7	
Middle school	41	22.8	35	19.8	
High school and diploma	62	34.4	58	32.9	
Academic	37	20.6	34	19.4	

* Chi-square test

According to the independent t-test before the educational intervention, there was no significant difference between the mean and standard deviation of dimensions of TPB (attitude, subjective norms (SN), perceived behavioral control (PBC) and behavioral intention), and AP and CP constructs in the experimental and control groups and the groups were also similar (*p* > 0.05).

After the intervention, the paired t-test indicated a significant difference between the mean and standard deviation of TPB and AP and CP constructs in the experimental group before and after the intervention (*p* < 0.05) (see Table 2). The analysis of covariance was used. It allows investigating differences between groups in the post-test simultaneously with statistical control of pre-test (covariate or covariate variable) in both groups. The research hypotheses indicated that the assumption of linearity and equality of variances was established, and the regression slope had homogeneity. Covariance analysis indicated a significant difference between scores of intervention and control groups under statistical control of post-test in two groups (covariate) after peer-led education (post-test) (*p <* 0.05). According to partial eta squared, 5.9 and 14% of the variance of each variable (attitude, SN, and intention) were due to the intervention effect (see Table 3).

**Table 2:** Comparison of the TPB construct scores between both groups before and after the intervention

Variable	GroupTime	Experimental group Mean ± SD	Control group Mean ± SD	***P-***value*
Attitude	Baseline	5.5 ± 19.0	4.9 ± 18.1	0.109
	2-months follow-up	6.2 ± 23.2	6.6 ± 18.4	0.001
	*P-*value**	0.001	0.777	–
*PBC*	Baseline	4.4 ± 13.4	3.7 ± 13.6	0.625
	2-months follow-up	5.2 ± 14.9	4.2 ± 13.7	0.037
	*P-*value**	0.006	0.933	–
SN	Baseline	1.9 ± 5.0	2.2 ± 4.7	0.160
	2-months follow-up	3.7 ± 6.9	2.5 ± 4.8	0.001
	*P-*value**	0.001	0.658	–
*AP*	Baseline	8.5 ± 4.5	4.5 ± 8.8	0.585
	2-months follow-up	6.3 ± 10.9	4.0 ± 8.7	0.001
	*P-*value**	0.001	0.991	–
CP	Baseline	6.9 ± 14.4	6.6 ± 14.7	0.970
	2-months follow-up	8.1 ± 16.6	6.7 ± 14.4	0.048
	*P-*value**	0.011	0.712	–
intention	Baseline	3.2 ± 6.0	2.7 ± 5.6	0.301
	2-months follow-up	4.1 ± 7.8	2.5 ± 5.0	0.001
	*P-*value**	0.001	0.008	

* Independent T-test; ** Paired T-test

**Table 3:** Covariance Analysis of TPB Variables, Action and Coping plan

Variable	Sum of Squares	df	Mean Square	F	Sig.	Partial Eta Squared	Noncent. Parameter	Observed Powera
Attitude	1878.8	1	1878.8	20.5	0.001	0.05	20.5	0.99
SN	364.1	1	364.1	36.5	0.001	0.09	36.5	1.0
PBC	206.5	1	206.5	7.9	0.005	0.02	7.9	0.80
AP	391.7	1	391.7	13.7	0.001	0.03	13.7	0.95
CP	463.7	1	463.7	7.4	0.007	0.02	7.4	0.77
Intention	567.6	1	567.6	59.4	0.001	0.14	59.4	1.0

In terms of tooth brushing behavior, only 24 (6.7%) students reported never having used toothbrushes before the intervention. After the intervention, the rate decreased to 10 (2.8%) students (10 children in the control group and 0 in the experimental group). Before the intervention, 76 students (21%) (40 children in the control group and 36 in the experimental group) brushed at least twice a day. After the intervention, the number increased to 128 (36%) (42 children in the control group and 86 in the experimental group). Differences between the two groups were statistically significant (p<.001).

When documenting flossing, 216 students (60.6%) (115 children in the control group and 111 in the experimental group) reported not using dental floss before the intervention but this decreased to 156 after the intervention. Over 70% of them (110 children) were in control group. Differences between the two groups were statistically significant (p<.001) (see Table 4).

**Table 4:** Comparison of the brushing and flossing behavior between both groups before and after intervention

Group	brushing	Experimental		Control		***P-***value*
Time of research		Number	Percentage (%)	Number	Percentage (%)	
Before intervention	never	13	7.2	11	6.2	0.678
	sometimes	66	36.7	55	31.3	
	once daily	65	36.1	70	39.7	
	two or more on a daily	36	20	40	22.8	
After intervention	never	0	0	10	5.7	0.001
	sometimes	15	8.4	51	28.9	
	once daily	79	43.8	73	41.6	
	two or more on a daily	86	47.8	42	23.8	
	*P-*value*	0.001		0.958		
Before intervention	Dental floss					***P-*value** **
	Yes	69	38.3	61	34.6	0.510
	No	111	61.7	115	65.4	
After intervention	Yes	134	74.4	66	37.5	0.001
	No	46	25.6	110	62.5	
	*P-*value**	0.013		0.657		

*chi-square **Fisher Exact test

## Discussion

Primary school aged students are an important group for interventions, as many self-regulated self-care behaviors are established at this stage in life. Our findings suggest that the group education approach merged with a planning intervention may lead to improved oral self-care behaviors such as brushing time, brushing technique, and flossing. This result is in line with previous findings [30, 43], stating that action and coping planning can facilitate oral hygiene behavior when individuals have control over their behavior. The result that PBC, action planning, and coping planning may predict oral self-care behavior is in accordance with previous studies [30, 44]. The comparison of mean scores for behavioral intention, AP, and CP before and after the intervention indicated that a significant difference between mean scores of experimental and control groups after the intervention. In other words, our results suggest that encouraging people to plan their time, place, and method for brushing could affect the initiation of consistent brushing behavior.

In the present study, two planning strategies, (CP and AP), were used to reduce the intention-behavior gap. AP facilitates the initiation of behavior and specifies how, where, and when the desired behavior takes place, while the AP predicts and tries to overcome obstacles and problems that may prevent the implementation of planned behavior. Consistent with findings of the present study, studies in Poland [45]. and China [46] indicate that two constructs (planning for action and coping) were important motivational variables for creating the OH behavior. Similarly with our study, we suggest these variables can be used as complementary for theory-based training programs.

In the experimental group, the number of students who reported never brushing decreased significantly from 13 and 66% and those reporting sometimes brushing from 0 and 15%. Furthermore, the use of dental floss almost doubled and this was statistically significant. Consistent with our findings, other researchers have found that peer-led approaches are more influential than the adult-led approaches in enhancing oral health-related behavior [47, 48], [49, 50].

We found that the positive attitude of children to brushing and dental flossing in the prevention of dental caries and periodontal diseases was enhanced among the peer-led health education groups suggesting a positive attitude to preserving appropriate oral health. The findings are in line with a nationally representative sample of Chinese adolescent students where a positive attitude about the prevention of dental caries and periodontal diseases was reported [51].

The present study shows that a short-term theory-based intervention that applied a planning component may improve OH behaviors (especially flossing) and is capable of preventing oral disease in primary school aged children. Our results showed increasing dental floss usage when students scheduled the time, place and method to complete the task of flossing. Other studies have shown that the flossing behavior of participants is enhanced when they have such plans [44, 52–54]. But barriers to effective implementation remain. To overcome this a coping program may help to facilitate the efficiency of an activity and decrease the impact of interference on positive OH behaviors. In our study participants in the intervention arm had higher levels of attitude, SN, and control beliefs than the control group. Therefore, we argue that the peer-led health education intervention can enhance benefits of planned behavior.

The education method was successful because students helped each other in the process of learning and the students were comfortable learning from their peers. Another possible reason for our success was the use of extended TPB. Previous studies mentioned the effectiveness of educational interventions by extended TPB [55, 56]. Results of our study indicated that attitude scores significantly increased in the experimental group after the intervention compared to the control group. Consistent with our findings, the improvement of attitude for the OH was also seen in other studies in similar settings including Bangladesh [57] and India [58]. Previous research indicated an increase or improvement in action planning for OH as key to achieving optimal behavior in the field of OH [59]. Experts believe that attitudes are obtained based on the individual experience of behavior or alternative experiences through learning from others; hence, as behavior is directly experienced, positive beliefs are strengthened in relation to the behavior outcome, and then act as incentives for its continuation [60].

Our findings were not unexpected and indicate that the educational intervention improved subjective norms in the experimental group. By using a peer-led model the increase in subjective norms was predictable and led to an increase in participation of students. We posit that students are more influenced by their peers and the school environment; and the school plays a key role in their oral health promotion. We found PBC as the most significant factor predicting oral hygiene behaviors; simultaneous control over barriers to apply the target behavior noticeably effected decisions considering behavior changes. In addition, when people recognize that behavioral factors have greater impact over their decisions it is also more likely that they perform relevant health behaviors. Previous studies of OH also indicate that the PBC was a strong determinant of behavioral intention [61]. Naseri and colleagues emphasized that health workers should plan and emphasize the promotion of PBC in order to design successful intervention measures for improving the OH because the higher PBC enhances a positive feeling about a desired behavior and reduces perceived barriers [62].

Our study was not without its limitations. First, the gender differences at baseline between the two arms of the study may impact the internal validity of the findings. Single-sex education in Iran effects the educational achievement of students but is just one dimension, other significant aspects are cultural, social, political, economic, and religious all of which may determine the achievement of students. Gender was accounted for in our multiple regression models. Second, we used a self-report questionnaire for data collection and social desirability particularly regarding dental floss use may have affected the responses. We did, however, verify floss use frequency by measuring the length of floss in the returned floss boxes, potentially reducing concerns regarding validity. Third, large confidence intervals (CIs) were obtained for brushing time, use of the modified Bass brushing technique, and dental flossing behavior. The precision of the point estimate for the study results may have been limited because of these large CIs. A larger sample is suggested for future studies. Finally, the participants were selected from one single city meaning that findings cannot be generalized to other settings and populations in Iran or more widely. Nevertheless, this study shows the potential effects of using a theory-based oral health education intervention to improve oral self-care behaviors in primary school aged children.

## Conclusion

In the present study, peer-led education was able to increase levels of OH behavior in grade 6 students. Due to a shortage of professional health workers in school educational settings, and the ease, usefulness and low-cost of this peer-led method, further exploration of ways to it roll-out should be considered as one way of improving and enhancing OH behavior.

## Supplementary information

**Additional file 1.** STROBE checklist. Checklist of items that should be included in reports of observational studies.

**Additional file 2.** English language version of the questionnaire.

## Data Availability

The datasets used and/or analyzed during the current study are available from the corresponding author on reasonable request.

## Abbreviations

AP: Action Plan
BS: Brain storming
CP: Coping Plan
DMFT: Decayed, Missing, and Filled Teeth
GD: Group discussion
OH: Oral health
PBC: Perceived behavioral control
SN: Subjective norms
TPB: Theory of planned behavior
WHO: World health organization
