# Supplementary material for: Oral health behavior among school children aged 11–13 years in Saveh, Iran: an evaluation of a theory-driven intervention
Source: BMC Pediatr. 2020 Oct 13;20:476. doi: 10.1186/s12887-020-02381-6 (PMC7552527; doi:10.1186/s12887-020-02381-6)
Supplement: Supplementary file 2 — Additional file 2. English language version of the questionnaire. [file 12887_2020_2381_MOESM2_ESM.docx]

**1.** How often do you brush your teeth?

1. Never b. less than once a month c. less than once a week d. about once a week

e. once a day f. twice a day or more

2.how often do you use dental floss?

1. Never b. less than once a month c. less than once a week d. about once a week

e. once a day f. twice a day or more

3.when was your last visit to the dentist?

a. never b. last year c. six months ago d. less than six months ago

4. What is the most important reason for you to go to the dentist?

A- For regular examination of my teeth B- Forcing Parents C- Having a toothache or a problem in the gums

Please circle the appropriate option:

5. How many teeth adults have?

a- 20 numbers b- 22 numbers c- 30 numbers d- 32 numbers

6. At what age do permanent teeth usually start to grow?

a- 3 years old b- 6 years old c- 9 years old d- 2 years’ old

7. what are the effects of fluoride on teeth?

a. Whitens teeth b. Increases tooth growth c - Prevents tooth decay d. Reduces tooth growth

8- Which of the following foods consumption causes tooth decay?

a. Foods containing sugars b. Foods containing protein such as meat

c- Fatty foods d. Fruits and vegetables

9. what is dental plaque?

a. It is a kind of toothpaste. b- It is a black spot on the surface of the tooth

c. Accumulation of germs and food particles that form on tooth surfaces. d- The food left between the teeth

10- What is the most important cause of bleeding gums when brushing?

A- Gum Disease B- Tooth Decay C- Tooth Plaque D- Improper brushing

11- -Which one is a sign of tooth decay?

A- The presence of brown or black spots on the teeth B- Sensitivity of teeth to cold or heat

C- Feeling of pain when chewing food D- All cases

12-how often do you change your brush?

A-1 to 3 months B-4 to 6 months C-8 to 10 months D-1 year

13-how many times we should brush our teeth in a day?

A - Once B - Twice

C- Three Times D- After waking up, after each meal and before going to bed

14. how long should it take to brush your teeth?

a. a minute b. two minutes c. three minutes d.4-5 minutes

15. How many times did you brush your teeth in the past two weeks? (please mention the number of it)

| Number | Items | Completely agree | agree | No idea | disagree | Completely disagree |
| --- | --- | --- | --- | --- | --- | --- |
| 16 | If I brush my teeth every day, I will suffer less from tooth decay. |  |  |  |  |  |
| 17 | If I brush my teeth every day, I will get less gum disease. |  |  |  |  |  |
| 18 | If I brush my teeth every day, I will have less toothache |  |  |  |  |  |
| 19 | If I brush every day, I will lose less teeth. |  |  |  |  |  |
| 20 | If I brush my teeth every day, I will have less heart disease in the future. |  |  |  |  |  |
| 21 | If I brush my teeth every day, my ability to chew food is preserved. |  |  |  |  |  |
| 22 | If I brush my teeth every day, my mouth will be fragrant |  |  |  |  |  |
| 23 | If I brush my teeth every day, my self-confidence will increase. |  |  |  |  |  |
| 24 | If I brush my teeth every day, my dental and medical expenses will be lower. |  |  |  |  |  |
| 25 | If I brush my teeth every day, my teeth will always be white. |  |  |  |  |  |
| 26 | If I brush every day, my smile looks more beautiful. |  |  |  |  |  |
| 27 | If I brush my teeth every day, the number of my visits to the dentist will be less. |  |  |  |  |  |
| 28 | Most people who are important to me (like my dad, my mom, my friends, my teacher, my dentist, etc.) think I should brush my teeth every day. |  |  |  |  |  |
| 29 | I think most people who are important to me (like my dad, my mom, my friends, my teacher, my dentist, etc.) brush their teeth every day. |  |  |  |  |  |
| 30 | My teachers encourage me to brush my teeth every day. |  |  |  |  |  |
| 31 | My friends encourage me to brush my teeth every day |  |  |  |  |  |
| 32 | If I want, it is easy for me to brush my teeth every day. |  |  |  |  |  |
| 33 | I'm sure I can brush my teeth every day |  |  |  |  |  |
| 34 | Even if I want to brush my teeth, I can't brush every day due to many factors (such as too much homework, boredom, forgetfulness, lack of time, etc.). |  |  |  |  |  |
| 35 | I think brushing is hard for me from now on |  |  |  |  |  |
| 36 | Brushing every day is completely under my control |  |  |  |  |  |
| 37 | I intend to brush my teeth every day for the next 2 weeks. |  |  |  |  |  |
| 38 | I have decided to brush my teeth every day for the next 2 weeks. |  |  |  |  |  |
| 39 | I plan to brush my teeth every day for the next 2 weeks. |  |  |  |  |  |
| 40 | I will brush my teeth every day for the next 2 weeks. |  |  |  |  |  |
| 41 | I plan on brushing time |  |  |  |  |  |
| 42 | I plan on brushing place |  |  |  |  |  |
| 43 | I plan a specific way to brush my teeth. |  |  |  |  |  |
| 44 | I plan on the time that I spend for brushing my teeth. |  |  |  |  |  |
| 45 | I plan on the times I brush my teeth. |  |  |  |  |  |
| 46 | - If there is a problem while brushing, I have a specific plan to resolve it |  |  |  |  |  |
| 47 | I have a specific plan for dealing with obstacles when brushing. |  |  |  |  |  |
| 48 | If I forget to brush my teeth, I plan what I have to do |  |  |  |  |  |
| 49 | I have a specific program to deal with toothache when brushing. |  |  |  |  |  |
| 50 | It is difficult to brush, I plan what I should do. |  |  |  |  |  |
| 51 | I know how to encourage myself to brush. |  |  |  |  |  |
| 52 | When are the right times to start brushing? |  |  |  |  |  |
| 53 | I have a specific program to deal with bleeding gums when brushing. |  |  |  |  |  |
